# Clinical results of an inactivated anti-*brucella* vaccine in combination with immunomodulators

**DOI:** 10.14202/vetworld.2020.758-763

**Published:** 2020-04-22

**Authors:** Fayssal Bouchemla, Valery Alexandrovich Agoltsov, Stepan Yuryevich Veselovsky, Sergey Vasilyevich Larionov, Olga Mikhaylovna Popova, Dmitry Valentinovich Krivenko

**Affiliations:** 1 Department of Animal Disease, Veterinarian and Sanitarian Expertise, Faculty of Veterinary Medicine, Vavilov Saratov State Agrarian University, Saratov, Russia; 2 Department of Food Technology, Faculty of Veterinary Medicine, Vavilov Saratov State Agrarian University, Saratov, Russia

**Keywords:** brucellosis, fosprenil, polypeptide C, split-conjugated vaccine against animal brucellosis

## Abstract

**Aim:**

The aim of the study was to obtain a vaccine against animal brucellosis having high immunogenic properties by carrying an evaluation of the effectiveness of split-conjugated animal brucellosis vaccine combined with fosprenil and polypeptide C as a molecular immunomodulatory adjuvant according to the results of serological studies of the blood of animals: Agglutination reaction, complement fixation, and rose Bengal sample.

**Materials and Methods:**

Eighteen calves of Holstein Friesians breed, aged 5 months, with a living weight of 100-150 kg, were divided into three groups of six animals each. All animals were healthy and they received a prophylactic vaccination against brucellosis. The dry split-conjugated vaccine against brucellosis in animals was dissolved in saline and for this purpose, 10 ml of saline was poured into the vaccine vial. Then the content was mixed, and afterward 1 ml was used per animal. Fosprenil was used at the rate of 1 kg of animal weight: 100 kg (calf weight) was multiplied by 0.05 (dose/1 kg of animal weight); 5 ml of fosprenil was obtained, which was collected into disposable syringes and intramuscularly sterilely injected into the croup area.

Calves in the first group (control) were intramuscularly injected with the vaccine at a dose of 1.0 ml, and fosprenil at a dose of 5.0 ml was administered intramuscularly once to the croup area. Animals from the second group were subcutaneously immunized by the vaccine with polypeptide C at a dose of 1.0 ml. Polypeptide C is a solution that was poured into a vial with a vaccine at a dose of 10.0 ml, the content was mixed, and then calves were injected subcutaneously into the middle third of the neck in 1 ml (10 doses in a vial).

Immunization of calves in the third group was carried out with a vaccine, diluted with an isotonic sodium chloride solution of 0.9%, at a dose of 1.0 ml subcutaneously once. At the 14^th^, 30^th^, and 90^th^ days after vaccination, a blood sampling was taken for serological tests: Agglutination test, complement fixation test, and rose Bengal test.

**Results:**

After conducting serological studies, it was noted that split-conjugated vaccine against animal brucellosis using fosprenil forms antibodies in large titers and they persist for a longer time in the body of animals compared to the other tested vaccine: The first combination with the immunomodulatory polypeptide C and the vaccine only on the physiological solution.

**Conclusion:**

The developed complex of split-conjugated vaccine against brucellosis in animals enhances the humoral immune response of the organism against brucellosis and improves the protection of animals against the disease when it is used with the immunomodulatory fosprenil. In the future, we want to expand the use of the resulting complex in the fight against brucellosis on a larger population and to study the change in cellular immunity after the introduction of the resulting complex on an animal organism.

## Introduction

Farm animals’ brucellosis is one of the most complicated themes in world veterinary science. The disease is common in Russia, Kazakhstan, Europe, as well as in African countries such as Algeria and others [1]. This disease causes significant economic damage to livestock farms, especially in cattle and sheep farming. Moreover, brucellosis infection poses a great threat to human health [2]. Although it is not classified as a conventional disease, brucellosis is nevertheless one of the ubiquitously distributed across the globe, representing a high degree of danger to people and animals [3,4]. *Brucella*, as an optional intracellular pathogen, establishes a close connection to the host immune cells. This pathogen is capable of supporting a chronic infection, which often complicates any treatment and diagnosis. However, it is worth mentioning that vaccination is the most economical measure to control brucellosis in the endemic areas. In recent decades, many studies have been carried out to develop a safe and effective vaccine against animal brucellosis. Till present, there is no licensed vaccine for the prevention of human brucellosis, which would be useful for protecting people who live in brucellosis endemic areas, especially farmers, veterinarians, animal care workers, laboratory personnel, and the general population [5]. Studies aimed at developing an ideal vaccine against animal and human brucellosis have been conducted since the beginning of the 20^th^ century [6]. Since then, inactivated and attenuated vaccines have been developed [7].

The Limited Liability Company Agrovet has developed a split-conjugated vaccine against animal brucellosis, containing proteins and soluble high molecular weight peptides of the vaccine strain, which is conjugated with the nano immunoprotector polypeptide-C and adsorbed onto the gel of *aluminum* hydroxide [8,9]. In laboratory animals, a polypeptide-C test showed its high ability to activate cellular and humoral mechanisms of immunity. This vaccine has the prospect of being used in veterinary practice and it will help to improve and support the animals’ well-being.

It was proven that fosprenil activates cellular immunity, in particular, systems of natural resistance (serum bactericidal activity and phagocytosis) and enhances the humoral immune response to a number of bacterial vaccines, thereby increasing the body’s resistance to infections [9]. Studies on the use of immunomodulatory in industrial poultry farming, such as imunofan, gamovit, and fosprenil, have shown their effectiveness in maintaining chicken population, increasing weight gain, as well as their immune activity. As a result of production experience, safety in the experimental groups was 100% against 96% of the control, the average weight in the experimental group reached 2960 g whereas in control 2490 g, while the intensity of immunity in the experimental group reached 80% as opposed to the control group: 65% [10]. The data presented of pharmacological action of the plant drug fosprenil indicate that fosprenil has a pronounced ability to optimize metabolic processes, provides the intensity of immunity against salmonellosis in piglets under conditions of increased technological load on their body [11].

At present, there is poor information about using fosprenil in any composition of anti-brucellosis vaccines. To use this immunomodulatory as a part of the anti-*Brucella* vaccine, with sufficiently high immunogenicity, the study of the fosprenil as a molecular immunomodulatory-adjuvant for split-conjugated brucellosis vaccine seems pertinent.

The split-conjugated vaccine against animal brucellosis is inactivated, which, according to some authors, nullify harmless, non-toxic both to animals and for the environment [12-16]. This allows the vaccine to be used at any time during pregnancy [9]. It is believed that the strains present in attenuated vaccines can be eliminated in the environment, restore their pathogenic properties and thereby maintain the local epizootic distress, not only for animals but can also become a source of infection for humans [13-16].

The aim of the study was to obtain a vaccine against animal brucellosis having high immunogenic properties by carrying an evaluation of the effectiveness of split-conjugated animal brucellosis vaccine combined with fosprenil and polypeptide C as a molecular immunomodulatory adjuvant according to the results of serological studies of the blood of animals: Agglutination reaction, complement fixation, and Rose Bengal sample.

## Materials and Methods

### Ethical approval

All studies involving calves were conducted according to the guidelines laid down by the European Convention for the Protection of Vertebrates used for the experimental and other scientific purposes, and in accordance with the local laws and regulations [17].

### Area and schema of the study

Studies were conducted in the period from June 1, 2018, to September 1, 2018. Under the observation, there were 18 calves of Holstein Friesians breed (male), aged 5 months, with a living weight of 100-150 kg and were divided into three groups of six animals each. The farm (LLC Berezovskoye), in which the experiment was carried out, is safe for animal brucellosis and this infection in this farm has never been registered.

The dry split-conjugated vaccine against brucellosis in animals was dissolved in saline and for this purpose, 10 ml of saline was poured into the vaccine vial. Then the content was mixed, and afterward 1 ml was used per animal.

Fosprenil was used at the rate of 1 kg of animal weight: 100 kg (calf weight) was multiplied by 0.05 (dose/1 kg of animal weight); and 5 ml of fosprenil was obtained, which was collected into disposable syringes and intramuscularly sterilely injected into the croup area.

Animals received a prophylactic vaccination against brucellosis. Calves in the first group (control) were intramuscularly injected with the vaccine at a dose of 1.0 ml, and the fosprenil application at a dose of 5.0 ml was administered intramuscularly once to the croup area. The second (first experimental) group of calves was subcutaneously immunized by the vaccine with the polypeptide C at a dose of 1.0 ml. Polypeptide C is a solution that was poured into a vial with a vaccine at a dose of 10.0 ml, the content was mixed, and then calves were injected subcutaneously into the middle third of the neck in 1 ml (10 doses in a vial).

An immunization of calves in the third (second experimental) group was subcutaneously carried out with the vaccine, diluted with an isotonic sodium chloride solution at 0.9% in a dose of 1.0 ml.

### Tests and samples collections

Before vaccination, 14, 30, and 90 days after vaccination, a blood sampling was taken for serological tests: Agglutination test (AT), complement fixation test (CFT), and rose Bengal test (RBT) [5,18,19].

Furthermore, according to the results of blood serological tests of three experimental groups of animals, it was determined in which of these groups antibodies (AT) had been detected and through which period of time and for how long these antibodies have been stored in the blood of animals what determines the vaccine effect with the immunomodulatory fosprenil, polypeptide C and saline.

## Results

When conducting serological studies before immunization, negative results were obtained in the agglutination reaction (AT), in the reaction of CFT and RBT.

When serological tests had been conducted in before the immunization, negative results were obtained in the RBT, CFT, and AT (there were no animals with brucellosis). The updated process was proceeded later at the 14^th^ and 30^th^ days after the immunization, finding is illustrated in Tables-1 and 2.

**Table-1:** The results of serological tests 14 days after vaccination (n=18).

Calf number	Preparation	Results of research		

		RBT	CFT	AT (IU)
17342	Polypeptide - C	Positive	Negative	Positive (100)
17366	Polypeptide - C	Positive	Negative	Positive (200)
17316	Polypeptide - C	Positive	Negative	Positive (400)
17336	Polypeptide - C	Positive	Negative	Positive (400)
17346	Polypeptide - C	Positive	Negative	Positive (200)
17278	Polypeptide - C	Positive	Negative	Positive (200)
17333	Fosprenil	*	*	*
17328	Fosprenil	Positive	Negative	Positive (100)
17368	Fosprenil	Positive	Negative	Positive (100)
17256	Fosprenil	Positive	Negative	Positive (100)
17312	Fosprenil	Positive	Negative	Positive (100)
17364	Fosprenil	Positive	Negative	Positive (100)
17306	Saline	Positive	Negative	Positive (50)
17320	Saline	Positive	Negative	Positive (100)
17310	Saline	Positive	Negative	Positive (200)
17274	Saline	Positive	Negative	Positive (100)
17248	Saline	Positive	Negative	Positive (50)
17334	Saline	Positive	Negative	Positive (100)

* Is not a correct result. *This is an animal that was transferred to another herd and initially we vaccinated it with vaccine, but further studies of the blood of this animal were not carried out due to the fact that it was removed from the studied herd. RBT=Rose Bengal test, CFT=Complement fixation test, AT=Agglutination test

**Table-2:** The results of serological studies 30 days after vaccination (n=18).

Calf number	Preparation	Results of research		

		RBT	CFT	AT (IU)
17342	Polypeptide - C	Doubtful	Negative	Negative
17366	Polypeptide - C	Negative	Negative	Negative
17316	Polypeptide - C	Positive	Positive (1/5)	Positive (50)
17336	Polypeptide - C	Positive	Positive (1/20)	Positive (50)
17346	Polypeptide - C	Positive	Positive (1/5)	Doubtful
17278	Polypeptide - C	Positive	Positive (1/5)	Doubtful
17333	Fosprenil	*	*	*
17328	Fosprenil	Negative	Negative	Negative
17368	Fosprenil	Positive	Positive (1/20)	Positive (50)
17256	Fosprenil	Positive	Positive (1/10)	Positive (50)
17312	Fosprenil	Negative	Negative	Negative
17364	Fosprenil	Negative	Negative	Negative
17306	Saline	Negative	Negative	Negative
17320	Saline	Positive	Positive (1/5)	Positive (50)
17310	Saline	Negative	Negative	Negative
17274	Saline	Positive	Positive (1/20)	Positive (50)
17248	Saline	Negative	Negative	Negative
17334	Saline	Positive	Positive (1/5)	Positive (50)

* Is not a correct result. RBT=Rose Bengal test, CFT=Complement fixation test, AT=Agglutination test

Table-1 represents the results of serological tests 14 days after the immunization against brucellosis in three different groups: The polypeptide C, fosprenil was added to the one and two experimental groups and, to the control group, the saline solution was injected. The given data indicate that by that time, there were no complementing antibodies in diagnostic titers. In the RBT and AT, all tests gave a positive result; however, the specific antibodies titers were different.

The highest titers were found in samples of serum calves, which were additionally injected with the polypeptide C (up to 400 IU). In samples, to which fosprenil was added, the antibodies’ titers kept the same level (100 IU). Antibodies titers in samples without immunomodulatory (saline) ranged from 50 to 200 IU. In the CFT, all samples had a negative result.

After 30 days of observation, the data obtained are presented in Table-2. The results of serological studies 30 days after immunization against brucellosis using the vaccine with polypeptide C, fosprenil, and to the control samples where saline solution was added are presented in Table-2.

Findings demonstrate that in the RBT, samples from the calves’ blood serum, which was additionally injected with polypeptide C had four positive cases out of six, one sample was doubtful and the other one was negative. The samples from calves, which were additionally administered fosprenil, had different results: Two cases out of six gave a positive result, one result was incorrect, and the other ones got a negative result. In samples of calves in the control group (saline), 50% of the samples revealed a positive result.

In the CFT, the titers of specific antibodies were varied even though not all the samples were positive: Four out of six in blood samples from calves, which were additionally injected with polypeptide C, had a positive result (from 1/5 to 1/20), one sample was doubtful, and one had a negative result. In tests with fosprenil, two samples were positive (from 1/10 to 1/20), three were negative, and one had an incorrect result. The control samples (saline) resulted in three positive out of six samples (from 1/5 to 1/20).

In the case of the RA, serum samples of calves, which had polypeptide C additionally injected with, got two positive samples (50 IU). In samples, where fosprenil was introduced, we found the same results. Apart of that, the antibodies’ titer in the control samples got three samples positive out of six (50 IU).

The results of serological tests in RBT, CFT, and AT, which were carried out 90 days after vaccination, indicated the absence of positive anti-Brucella results in serological reactions.

## Discussion

According to some authors who used fosprenil as an immunomodulatory in chickens, it was found that this drug increases the concentration of immunoglobulins in broiler blood; in particular, there is an increase in the concentration of specific antibodies [20], which was evidenced by the results of our research.

The obtained data from Melnik’s study have demonstrated the efficacy of fosprenil use in vaccine industry. It was revealed in the same study (Melnik. 2013) that the active ingredient of forprenil is disodium salt of phosphate polyprenols. The introduction of the drug in a dose of 2.5 ml once a day for 5 days activates metabolic processes and, accordingly, increases the average daily weight gain [21]. In the experimental group, where the immunostimulant was injected, no cases of bronchopneumonia were recorded for 2 months of observation. Under the influence of the drug, morpho-biochemical blood parameters increase within the physiological norm, and in calves of the control group, changes characteristic of the onset of the disease with bronchopneumonia were recorded. It follows from the above that fosprenil stimulates natural resistance, increases the body’s resistance to infections, and reduces the morbidity [21]. The data obtained by him have been confirmed in our research. Fosprenil also affects the weight gain of the bird, and contributes to the formation of bird immunity, as shown in the experiment on chickens, where the increase in chicken’s weight using fosprenil is greater than the weight of the control groups in which no fosprenil was administered [22].

In contrast to our experiment, the tested vaccine with immunomodulatory (polypeptide C and fosprenil) also enhances the humoral immune response. Our previous experiment (vaccine tested, and with polyoxidonium immunomodulatory) also showed an increase in humoral immunity in animals [23]. Furthermore, fosprenil is used in conjunction with other vaccines. It was shown that fosprenil significantly increased the specific protective activity of the rabies vaccine: When administered together, the immunization index increased 1.9 times as compared to the immunization scheme without fosprenil. In addition, the comparative immunogenicity index of the vaccine was 1.6 times higher with the vaccination using fosprenil. Thus, the indicated finding demonstrates that fosprenil is able to potentiate the specific immunogenicity of tick-borne encephalitis and rabies vaccines [24].

Experimental data indicate the possibility of using fosprenil for the prevention of avian influenza in combination with vaccination and quarantine measures – to create a reliable barrier against infection [25].

The results have shown that split-conjugated vaccine against animal brucellosis is a promising drug for the prevention of this zoonosis.

The complement-binding antibodies were not detected by serological tests 14 days after immunization; however, in the AT and in the RBT, all samples were positive while highest values in AT (up to 400 IU) were observed in samples of calves’ blood, which were injected polypeptide C.

On the 30^th^ day, after immunization in the CFT, not all samples were positive. Using the RBT, samples, to which polypeptide C was injected, had a positive result of 67%. Whereas samples, where fosprenil was injected, a positive result was noted just in two samples as opposed to the control group (saline) where 50% of them gave a positive result.

The results of serological studies conducted 90 days after vaccination indicate that all animals with a serological reaction negatively responded to brucellosis.

## Conclusion

Despite the small number of animals in the experiment, it was found that the tested, which is considering as an inactivated vaccine, forms a specific humoral immunity in animals. It has tremendous advantages over currently used live vaccines, the causative agent of which is able to survive in the environment, thereby participating in the spread of animal and human brucellosis.

The developed complex of the split-conjugated vaccine against brucellosis in animals enhances the humoral immune response of the organism against brucellosis and improves the protection of animals against the disease when it is used with the immunomodulatory fosprenil. In the future, we want to expand the use of the resulting complex in the fight against brucellosis on a larger population and to study the change in cellular immunity after the introduction of the resulting complex on an animal organism.

## Authors’ Contributions

VAA and FB designed the work. All authors conducted the research work. FB, SYV, OMP, DVK, and SVL analyzed the data analysis and drafted the manuscript under the guidance of VAA. All authors read and approved the final manuscript.
